# Long-term effect of hospital volume on the postoperative prognosis of 158,618 patients with esophageal squamous cell carcinoma in China

**DOI:** 10.3389/fonc.2022.1056086

**Published:** 2023-02-16

**Authors:** Ling-Ling Lei, Xin Song, Xue-Ke Zhao, Rui-Hua Xu, Meng-Xia Wei, Lin Sun, Pan-Pan Wang, Miao-Miao Yang, Jing-Feng Hu, Kan Zhong, Wen-Li Han, Xue-Na Han, Zong-Min Fan, Ran Wang, Bei Li, Fu-You Zhou, Xian-Zeng Wang, Li-Guo Zhang, Qi-De Bao, Yan-Ru Qin, Zhi-Wei Chang, Jian-Wei Ku, Hai-Jun Yang, Ling Yuan, Jing-Li Ren, Xue-Min Li, Li-Dong Wang

**Affiliations:** ^1^ State Key Laboratory of Esophageal Cancer Prevention and Treatment and Henan Key Laboratory for Esophageal Cancer Research of The First Affiliated Hospital, Zhengzhou University, Zhengzhou, Henan, China; ^2^ Department of Thoracic Surgery, Anyang Tumor Hospital, Anyang, Henan, China; ^3^ Department of Thoracic Surgery, Linzhou People’s Hospital, Linzhou, Henan, China; ^4^ Department of Thoracic Surgery, Xinxiang Central Hospital, Xinxiang, Henan, China; ^5^ Department of Oncology, Anyang District Hospital, Anyang, Henan, China; ^6^ Department of Oncology, The First Affiliated Hospital of Zhengzhou University, Zhengzhou, Henan, China; ^7^ Department of Gastroenterology, The Second Affiliated Hospital of Nanyang Medical College, Nanyang, Henan, China; ^8^ Department of Pathology, Anyang Tumor Hospital, Anyang, Henan, China; ^9^ Department of Radiotherapy, The Affiliated Cancer Hospital of Zhengzhou University (Henan Cancer Hospital), Zhengzhou, Henan, China; ^10^ Department of Pathology, Second Affiliated Hospital of Zhengzhou University, Zhengzhou, China; ^11^ Department of Pathology, Hebei Provincial Cixian People’s Hospital, Cixian, Hebei, China

**Keywords:** hospital volume, esophageal squamous cell carcinoma, esophagectomy, postoperative survival, retrospective analysis

## Abstract

**Background:**

The impact of hospital volume on the long-term survival of esophageal squamous cell carcinoma (ESCC) has not been well assessed in China, especially for stage I–III stage ESCC. We performed a large sample size study to assess the relationships between hospital volume and the effectiveness of ESCC treatment and the hospital volume value at the lowest risk of all-cause mortality after esophagectomy in China.

**Aim:**

To investigate the prognostic value of hospital volume for assessing postoperative long-term survival of ESCC patients in China.

**Methods:**

The date of 158,618 patients with ESCC were collected from a database (1973–2020) established by the State Key Laboratory for Esophageal Cancer Prevention and Treatment, the database includes 500,000 patients with detailed clinical information of pathological diagnosis and staging, treatment approaches and survival follow-up for esophageal and gastric cardia cancers. Intergroup comparisons of patient and treatment characteristics were conducted with the X^2^ test and analysis of variance. The Kaplan-Meier method with the log-rank test was used to draw the survival curves for the variables tested. A Multivariate Cox proportional hazards regression model was used to analyze the independent prognostic factors for overall survival. The relationship between hospital volume and all-cause mortality was assessed using restricted cubic splines from Cox proportional hazards models. The primary outcome was all-cause mortality.

**Results:**

In both 1973-1996 and 1997-2020, patients with stage I-III stage ESCC who underwent surgery in high volume hospitals had better survival than those who underwent surgery in low volume hospitals (both P<0.05). And high volume hospital was an independent factor for better prognosis in ESCC patients. The relationship between hospital volume and the risk of all-cause mortality was half-U-shaped, but overall, hospital volume was a protective factor for esophageal cancer patients after surgery (HR<1). The concentration of hospital volume associated with the lowest risk of all-cause mortality was 1027 cases/year in the overall enrolled patients.

**Conclusion:**

Hospital volume can be used as an indicator to predict the postoperative survival of ESCC patients. Our results suggest that the centralized management of esophageal cancer surgery is meaningful to improve the survival of ESCC patients in China, but the hospital volume should preferably not be higher than 1027 cases/year.

**Core tip:**

Hospital volume is considered to be a prognostic factor for many complex diseases. However, the impact of hospital volume on long-term survival after esophagectomy has not been well evaluated in China. Based on a large sample size of 158,618 ESCC patients in China spanning 47 years (1973-2020), We found that hospital volume can be used as a predictor of postoperative survival in patients with ESCC, and identified hospital volume thresholds with the lowest risk of death from all causes. This may provide an important basis for patients to choose hospitals and have a significant impact on the centralized management of hospital surgery.

## Introduction

Esophageal cancer is the seventh most common malignant tumor (604,100 new cases in 2020) and the sixth deadliest tumor (544,000 deaths in 2020) in the world (1, 2). With the development of the economy and the increase in people’s health consciousness, most patients with esophageal cancer prefer to choose medium volume or high volume hospitals instead of low volume hospitals in China. For hospitals, doctors and patients, hospital volume has been recognized as an important determinant of patient survival (3, 4). Halm et al. found that admission to higher-volume hospitals was associated with a reduction in mortality for many surgical conditions and medical procedures (5). Several studies have also showed that patients with esophageal cancer who received treatment in higher volume hospitals had significantly better long-term survival rates than patients treated at lower volume hospitals (4, 6–8). However, several other studies found that the hospital volume is not an important predictor of survival in esophageal cancer, nor should it be used as an alternative measure of surgical quality (9, 10). To better understand the relationship between hospital volume and the effectiveness of treatment in China, we analyzed the mortality and survival of 158,618 stage I–III patients with ESCC who underwent esophagectomy at different volume hospitals.

## Materials and methods

### Patients

A total of 158,618 patients who diagnosed as ESCC between 1973 and 2020 from the 500,000 esophageal and gastric cardia carcinoma databases (1973–2020), established by The State Key Laboratory for Esophageal Cancer Prevention and Treatment, were enrolled in this retrospective study (11–14). Patients were selected according to the following criteria: (1) Patients were diagnosed with ESCC by gastroscopy biopsy or postoperative histopathology. (2) Patients had no other malignant tumors except for ESCC. (3) Patients had a clear diagnosis time and underwent surgery only (patients with minimally invasive resection and preoperative and postoperative chemoradiotherapy were excluded). (4) Patients have complete clinical records. All medical records were reviewed for consistency and completeness.

### Hospital volume

**Figure 1:**
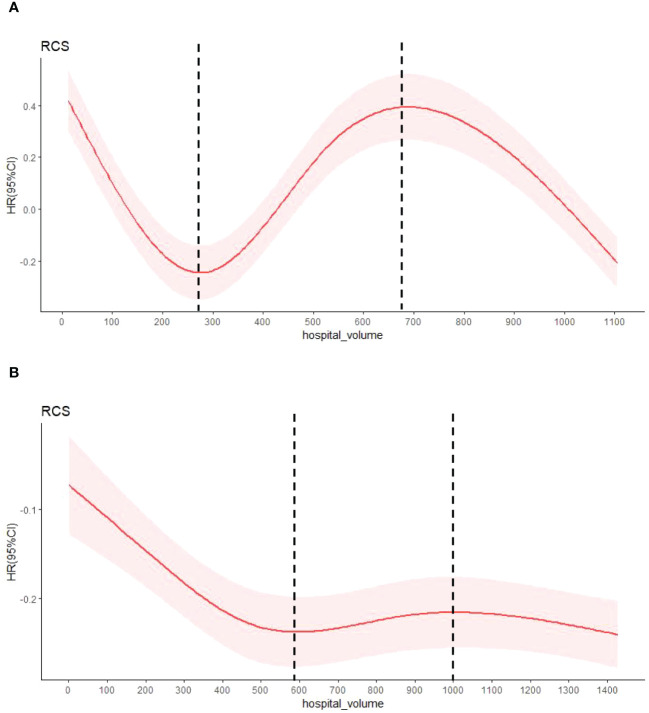
The HR by annual average hospital volume with restricted cubic spline fit. **(A)** shows patients with esophageal cancer from 1973 to 1996, and **(B)** from 1997 to 2020.The relationship between known covariable-adjusted risk of death and annual hospital volume. The solid red line represents a restricted cubic spline (RCS) fit and the light red shadow represents a 95% confidence interval for the RCS fit. The vertical dashed lines are the extremes of the curve.

Hospital volume was defined as the annual average number of esophagectomy procedures per hospital. To determine hospital volume groups, we created a multivariate Cox proportional hazards model with restricted cubic splines (RCS, **Figure 1**). The covariates in the model included sex, age, region, urban/rural residence, smoking history, drinking history, cancer family history, incisal edge residue, tumor location, differentiation and pathological stage. The RCS can explain the nonlinear relationship between the average annual hospital volume and survival rate, combined with the change in hazard ratio (HR), and the two extreme points of the curve are finally determined (in 1973-1996: 276.638 and 688.573; in 1997-2020: 596.181 and 1004.919). All hospitals were divided into low volume (1-277 cases/years and 1-596 cases/years), medium volume (278-689 cases/years and 597-1004 cases/years) and high volume (690-1106 cases/yearsand 1005-1428 cases/years) groups according to two integer extreme points.

### High/Low incidence area

Based on the epidemiological findings of esophageal cancer, the age and mortality rate were adjusted to include ESCC mortality rates of more than 60 per 100,000 is recognized as an ESCC high incidence area, while the others are a low incidence area. Zoning reference to 《esophageal cancer》.

### Urban/Rural residence

Those living in county level and above were classified as urban residents, while the rest were classified as rural residents.

### Smoking and alcohol consumption history

Smoking consumption history refers to smoking more than 1 cigarette per day, continuous or cumulative smoking for more than 6 months in a lifetime.

Alcohol consumption history refers to according to the record of excessive drinking, more than 4 standard cups (A standard cup is a drink containing 18 milliliters of alcohol.) per day and drinking more than 3 times a week.

### Family history of cancer

A positive family history of cancer is 2 or more cancer patients in the same family within consecutive 3 generations.

A negative family history of cancer means that only one patient with cancer in the same family within consecutive 3 generations.

### Treatment

Refer to the NCCN guidelines for the 1st edition of esophageal cancer in 2015 (15), and this study only included patients undergoing surgery of ESCC. The surgical methods mainly include Sweet procedure, Ivor-Lewis procedure, Mckeown procedure and transhiatal esophagectomy. Because transhiatal esophagectomy is rarely used in China, only Sweet procedure, Ivor-Lewis procedure and Mckeown procedure were considered in the surgical approach analysis in this study.

### Tumor staging

The time span of diagnosis of ESCC patients in this study was large, pathological staging of esophageal cancer has been updated in different editions (the sixth edition in 2002, the seventh edition in 2009, and the eighth edition in 2017).In order to reduce the error, the TNM staging of esophageal and esophagogastric junction cancer, the sixth edition jointly published by the International Union Against Cancer (UICC) and the American Cancer Federation (AJCC), was uniformly used in this study (16).

### Follow-up

The study follow-up was mainly carried out by correspondence, telephone calls, home visits and direct contact between village doctors and patients or their families or through systems such as the new cooperative medical database, the Medical Security Administration database and the registration and management of citizen death information. In 2 years after discharge, the patients were followed up every 3 months. Once every six months for 3-5 years. Then, follow-up was conducted once a year and until death, emigration, or the end of the study period (January 2021), whichever occurred first. Of the 158,618 ESCC patients 103,252 patients (65.1%) were followed-up successfully.

### Statistical analysis

Statistical analysis was performed using SPSS(Windows version 21.0) and R. The t test and chi-square test were used to compare the differences in categorical and continuous variables, respectively, between different ESCC groups. The survival outcome was estimated by the Kaplan-Meier method and the multivariate Cox proportional hazards regression model. Multivariate analysis adjusted for sex, age, region, urban/rural residence, smoking history, drinking history, cancer family history, incisal edge residue, tumor location, differentiation, pathological stage and diagnosis time. A value of *P*< 0.05 was considered statistically significant.

The association between hospital volume and all-cause mortality was assessed on a continuous scale using restricted cubic splines based on the Cox proportional hazards model. To balance best-fit and overfitting on the main splines of mortality, the Akaike information criterion was used to selects the number of knots between 3 and 7 as the lowest value, but if the number of different knots is within two, the lowest number was chosen. The hospital volume associated with the lowest risk of death was the value of the lowest hazard ratio on the spline curve.

## Results

### Patient eligibility

**Figure 2:**
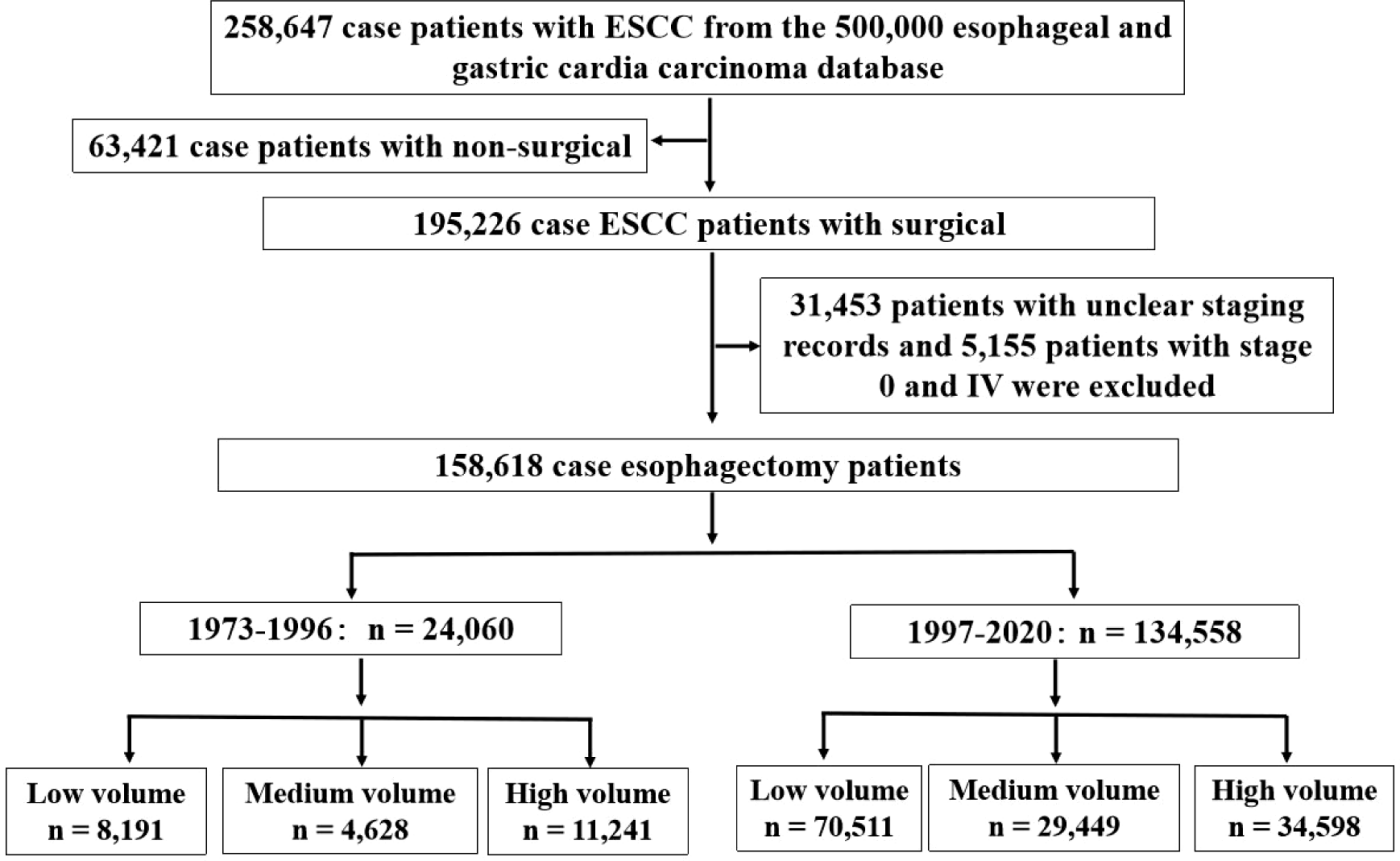
Study population selection criteria from database. The study population was selected form the 500,000 esophageal and gastric cardia carcinoma database (1973-2020) and included patients who were diagnosed ESCC from 1973 to 2020. The final cohort sample size was 158,618.

A total of 258,647 patients with ESCC were evaluated for eligibility. Of these 63,421 patients were excluded due to nonsurgical reasons. In addition, 31,453 patients with unclear staging records and 5,155 patients with stage 0 and IV were excluded. A total of 158,618 patients with ESCC were included, including 24,060 cases were diagnosed between 1973 and 1996, and 134,558 cases between 1997 and 2020. The 24,060 patients were from 38 hospitals, including 28 low volume hospitals, 7 medium volume hospitals and 3 high volume hospitals, and the 134,558 patients were from 101 hospitals, including 73 low volume hospitals, 21 medium volume hospitals and 7 high volume hospitals (**Figure 2**).

### Population demographics

From the archived clinical records, we retrieved the clinicopathological features of ESCC patients in this study during two time periods (**Tables 1**, **2**). In both time periods, the patients were mainly male (1973-1996:59.8%, 1997-2020:65.9%), 50-70 years old (1973-1996:67.4%, 1997-2020:75.2%), high incidence area (1973-1996:81.1%, 1997-2020:57.4%) and rural residents (1973-1996:89.5%, 1997-2020:88.5%). Nearly half of the patients in two groups had a positive family history of cancer (1973-1996:49.0%, 1997-2020:41.0%). In addition, almost all female patients had no cigarette smoking and alcohol consumption. In contrast, nearly 60 percent of male patients had a history of cigarette smoking (1973-1996:63.9%, 1997-2020:66.2%) and 40 percent had a history of alcohol consumption(1973-1996:36.6%, 1997-2020:45.7%). Almost two-thirds of the patients were diagnosed with stage III, 6.3% and 9.7% of patients with stage I underwent surgery at two time periods, respectively. The stage III patients from 1997 to 2020 were significantly higher than those from 1973 to 1996 (72.1% *vs*. 66.6%), and the positive rate of incisal edge residue was significantly lower than those from 1973 to 2020 (3.6% *vs*. 6.2%). In all patients with clear surgical approach records, left thoracotomy was the main method in both time periods(1973-1996:93.9%, 1997-2020:80.1%). Two-thirds of the tumor was located in the middle chest and were moderate differentiation. There were 5,631 postoperative complications, the most common of which were pulmonary complications(22.0%), anastomotic leak(20.3%) and incision infection(18.5%), the next were cardiovascular complications(10.1%), chylothorax(2.1%), recurrent laryngeal nerve injury(1.8%), etc., while surgical death(0.9%) and hoarseness(0.6%) were rare. Relapse was recorded in 2,673 patients, most of whom received radiotherapy or chemotherapy. The 1-year, 3-year, 5-year and 7-year survival rates of patients at both time periods were 83.4%, 69.8%, 59.3%, 11.4%(1973-1996) and 76.3%, 58.4%, 47.4%, 24.5(1997-2020), respectively.

**Table 1:** Relationship between the clinicopathological features of ESCC patients and hospital volume during 1973-1996, n(%).

Characteristics	No. of The Patients Examined		Hospital Volume						
			Low		Medium		High		P
Sex									0.026
Male	14390	(59.8)	4836	(59.0)	2844	(61.5)	6710	(59.7)	
Female	9670	(40.2)	3355	(41.0)	1784	(38.5)	4531	(40.3)	
Total	24060	(100.0)	8191	(100.0)	4628	(100.0)	11241	(100.0)	
Age									0.000
<40	1275	(5.3)	496	(6.1)	284	(6.1)	495	(4.4)	
40-	5572	(23.2)	2143	(26.2)	1142	(24.7)	2287	(20.3)	
50-	9380	(39.0)	3329	(40.6)	1910	(41.3)	4141	(36.8)	
60-	6840	(28.4)	2032	(24.8)	1177	(25.4)	3631	(32.3)	
70-	993	(4.1)	191	(2.3)	115	(2.5)	687	(6.1)	
Total	24060	(100.0)	8191	(100.0)	4628	(100.0)	11241	(100.0)	
Regions									0.000
HIA	19514	(81.1)	6768	(82.6)	3056	(66.0)	9690	(86.2)	
LIA	4546	(18.9)	1423	(17.4)	1572	(34.0)	1551	(13.8)	
Total	24060	(100.0)	8191	(100.0)	4628	(100.0)	11241	(100.0)	
Urban/Rural Residence									0.000
Urban	2524	(10.5)	965	(11.8)	422	(9.1)	1137	(10.1)	
Rural	21536	(89.5)	7226	(88.2)	4206	(90.9)	10104	(89.9)	
Total	24060	(100.0)	8191	(100.0)	4628	(100.0)	11241	(100.0)	
Cigarette Smoking									0.000
Yes	9626	(40.0)	3218	(39.3)	1979	(42.8)	4429	(39.4)	
No	14434	(60.0)	4973	(60.7)	2649	(57.2)	6812	(60.6)	
Total	24060	(100.0)	8191	(100.0)	4628	(100.0)	11241	(100.0)	
Alcohol Consumption									0.000
Yes	5722	(23.8)	2237	(27.3)	676	(14.6)	2809	(25.0)	
No	18338	(76.2)	5954	(72.7)	3952	(85.4)	8432	(75.0)	
Total	24060	(100.0)	8191	(100.0)	4628	(100.0)	11241	(100.0)	
Cancer Family History									0.000
Positive	11790	(49.0)	2481	(30.3)	1645	(35.5)	7664	(68.2)	
Negative	12270	(51.0)	5710	(69.7)	2983	(64.5)	3577	(31.8)	
Total	24060	(100.0)	8191	(100.0)	4628	(100.0)	11241	(100.0)	
Tumor Location#									0.000
Upper	4039	(16.9)	1042	(12.7)	1497	(32.5)	1500	(13.5)	
Middle	14429	(60.3)	5109	(62.4)	2186	(47.4)	7134	(64.1)	
Lower	5461	(22.8)	2039	(24.9)	930	(20.2)	2492	(22.4)	
Total	23929	(100.0)	8190	(100.0)	4613	(100.0)	11126	(100.0)	
Differentiation									0.000
Well	5353	(26.1)	2364	(34.2)	672	(16.3)	2317	(24.4)	
Moderate	11223	(54.7)	3383	(49.0)	2751	(66.7)	5089	(53.6)	
Poor	3949	(19.2)	1158	(16.8)	700	(17.0)	2091	(22.0)	
Total	20525	(100.0)	6905	(100.0)	4123	(100.0)	9497	(100.0)	
Incisal Edge Residue									0.000
Negative	13772	(93.8)	4270	(93.0)	3428	(98.8)	6074	(91.7)	
Positive	913	(6.2)	321	(7.0)	43	(1.2)	549	(8.3)	
Total	14685	(100.0)	4591	(100.0)	3471	(100.0)	6623	(100.0)	
Lymph Node Metastasis									0.000
Negative	14081	(58.5)	4544	(55.5)	2826	(61.1)	6711	(59.7)	
Positive	9979	(41.5)	3647	(44.5)	1802	(38.9)	4530	(40.3)	
Total	24060	(100.0)	8191	(100.0)	4628	(100.0)	11241	(100.0)	
Pathological Stage									0.000
I	1523	(6.3)	911	(11.1)	199	(4.3)	413	(3.7)	
II	6516	(27.1)	1500	(18.3)	1930	(41.7)	3086	(27.5)	
III	16021	(66.6)	5780	(70.6)	2499	(54.0)	7742	(68.9)	
Total	24060	(100.0)	8191	(100.0)	4628	(100.0)	11241	(100.0)	
Surgical Approaches$									0.000
Left	6601	(93.9)	1725	(99.7)	553	(98.8)	4323	(91.3)	
Right	426	(6.1)	6	(0.3)	7	(1.2)	413	(8.7)	
Total	7027	(100.0)	1731	(100.0)	560	(100.0)	4736	(100.0)	
Operative Deaths*									0.000
Yes	29	(0.1)	7	(0.1)	15	(0.3)	7	(0.1)	
No	24031	(99.9)	8184	(99.9)	4613	(99.7)	11234	(99.9)	
Total	24060	(100.0)	8191	(100.0)	4628	(100.0)	11241	(100.0)	
Death in Hospital&									0.000
Yes	89	(0.4)	23	(0.3)	35	(0.8)	31	(0.3)	
No	23971	(99.6)	8168	(99.7)	4593	(99.2)	11210	(99.7)	
Total	24060	(100.0)	8191	(100.0)	4628	(100.0)	11241	(100.0)	

HIA, high incidence area; LIA, low incidence area.

#:Because of the small number, cervical esophageal cancer was divided into the upper segment.

$:Left, Sweet procedure. Right: Ivor-Lewis procedure+Mckeown procedure.

*: Operative Death: Death within 14 days of esophagectomy or death during the hospitalization in which the primary procedure was performed.

&: Death in Hospital: Death within the same hospital admission or within 30 days.

**Table 2:** Relationship between the clinicopathological features of ESCC patients and hospital volume during 1997-2020, n(%).

Characteristics	No. of The Patients Examined		Hospital Volume						
	Patients Examined		Low		Medium		High		P
Sex									0.000
Male	88674	(65.9)	47692	(67.6)	20027	(68.0)	20955	(60.6)	
Female	45884	(34.1)	22819	(32.4)	9422	(32.0)	13643	(39.4)	
Total	134558	(100.0)	70511	(100.0)	29449	(100.0)	34598	(100.0)	
Age									0.000
<40	1244	(0.9)	761	(1.1)	233	(0.8)	250	(0.7)	
40-	12703	(9.4)	7146	(10.1)	2614	(8.9)	2943	(8.5)	
50-	46388	(34.5)	24920	(35.3)	10136	(34.4)	11332	(32.8)	
60-	54821	(40.7)	27972	(39.7)	12473	(42.4)	14376	(41.6)	
70-	19402	(14.4)	9712	(13.8)	3993	(13.6)	5697	(16.5)	
Total	134558	(100.0)	70511	(100.0)	29449	(100.0)	34598	(100.0)	
Regions									0.000
HIA	77272	(57.4)	36560	(51.9)	10958	(37.2)	29754	(86.0)	
LIA	57286	(42.6)	33951	(48.1)	18491	(62.8)	4844	(14.0)	
Total	134558	(100.0)	70511	(100.0)	29449	(100.0)	34598	(100.0)	
Urban/Rural Residence									0.000
Urban	15433	(11.5)	7655	(10.9)	4445	(15.1)	3333	(9.6)	
Rural	119125	(88.5)	62856	(89.1)	25004	(84.9)	31265	(90.4)	
Total	134558	(100.0)	70511	(100.0)	29449	(100.0)	34598	(100.0)	
Cigarette Smoking									0.000
Yes	60740	(45.1)	31959	(45.3)	13825	(46.9)	14956	(43.2)	
No	73818	(54.9)	38552	(54.7)	15624	(53.1)	19642	(56.8)	
Total	134558	(100.0)	70511	(100.0)	29449	(100.0)	34598	(100.0)	
Alcohol Consumption									0.000
Yes	41987	(31.2)	21614	(30.7)	9561	(32.5)	10812	(31.3)	
No	92571	(68.8)	48897	(69.3)	19888	(67.5)	23786	(68.7)	
Total	134558	(100.0)	70511	(100.0)	29449	(100.0)	34598	(100.0)	
Cancer Family History									0.000
Positive	55152	(41.0)	22827	(32.4)	8649	(29.4)	23676	(68.4)	
Negative	79287	(59.0)	47565	(67.6)	20800	(70.6)	10922	(31.6)	
Total	134439	(100.0)	70392	(100.0)	29449	(100.0)	34598	(100.0)	
Tumor Location#									0.000
Upper	21831	(17.0)	11370	(17.2)	3971	(14.3)	6490	(18.9)	
Middle	82543	(64.3)	41492	(62.7)	19048	(68.4)	22003	(64.0)	
Lower	24019	(18.7)	13286	(20.1)	4844	(17.4)	5889	(17.1)	
Total	128393	(100.0)	66148	(100.0)	27863	(100.0)	34382	(100.0)	
Differentiation									0.000
Well	19026	(15.2)	12712	(19.5)	3076	(11.6)	3238	(9.7)	
Moderate	77673	(62.1)	40736	(62.6)	16410	(61.7)	20527	(61.3)	
Poor	28451	(22.7)	11639	(17.9)	7105	(26.7)	9707	(29.0)	
Total	125150	(100.0)	65087	(100.0)	26591	(100.0)	33472	(100.0)	
Incisal Edge Residue									0.000
Negative	105773	(96.4)	54813	(97.3)	23880	(97.3)	27080	(93.8)	
Positive	3980	(3.6)	1524	(2.7)	659	(2.7)	1797	(6.2)	
Total	109753	(100.0)	56337	(100.0)	24539	(100.0)	28877	(100.0)	
Lymph Node Metastasis									0.000
Negative	80312	(59.7)	42192	(59.8)	17787	(60.4)	20333	(58.8)	
Positive	54245	(40.3)	28318	(40.2)	11662	(39.6)	14265	(41.2)	
Total	134557	(100.0)	70510	(100.0)	29449	(100.0)	34598	(100.0)	
Pathological Stage									0.000
I	13111	(9.7)	6432	(9.1)	3017	(10.2)	3662	(10.6)	
II	24377	(18.1)	12830	(18.2)	5297	(18.0)	6250	(18.1)	
III	97070	(72.1)	51249	(72.7)	21135	(71.8)	24686	(71.4)	
Total	134558	(100.0)	70511	(100.0)	29449	(100.0)	34598	(100.0)	
Surgical Approaches$									0.000
Left	33157	(80.1)	7612	(63.8)	8277	(93.7)	17268	(83.6)	
Right	8260	(19.9)	4314	(36.2)	553	(6.3)	3393	(16.4)	
Total	41417	(100.0)	11926	(100.0)	8830	(100.0)	20661	(100.0)	
Operative Deaths*									0.554
Yes	286	(0.2)	155	(0.2)	55	(0.2)	76	(0.2)	
No	134272	(99.8)	70356	(99.8)	29394	(99.8)	34522	(99.8)	
Total	134558	(100.0)	70511	(100.0)	29449	(100.0)	34598	(100.0)	
Death in Hospital&									0.665
Yes	734	(0.5)	380	(0.5)	155	(0.5)	199	(0.6)	
No	133824	(99.5)	70131	(99.5)	29294	(99.5)	34399	(99.4)	
Total	134558	(100.0)	70511	(100.0)	29449	(100.0)	34598	(100.0)	

HIA, high incidence area; LIA, low incidence area.

#:Because of the small number, cervical esophageal cancer was divided into the upper segment.

$:Left: Sweet procedure. Right: Ivor-Lewis procedure+Mckeown procedure.

*: Operative Death: Death within 14 days of esophagectomy or death during the hospitalization in which the primary procedure was performed.

&: Death in Hospital: Death within the same hospital admission or within 30 days.

### Univariate intergroup analysis by hospital volume

From 1973 to 1996, there were 14,390 male patients with a mean age of 54.5 ± 9.3 years and 9,670 female patients with a mean age of 54.8 ± 9.0 years. Individuals presenting to high volume hospitals were mostly from high incidence areas, more likely to be older at diagnosis and a positive family history of cancer, and had more stage III patients than the other two subgroups. The percentage of positive incisal edge residue was lowest in medium volume hospitals (1.2%), but the operative death and in-hospital death were both higher. A total of 426 ESCC patients underwent right thoracotomy, of which 413 (96.9%) were in high volume hospitals, 6 case(1.4%) were in low volume hospitals, and 7 case(1.6%) were in medium volume hospitals (**Table 1**).

From 1997 to 2020, there were 88,674 male patients with a mean age of 60.0 ± 8.5 years and 45,884 female patients with a mean age of 60.8 ± 8.4 years. There was no significant difference in operative death and in-hospital death among hospitals with different volume. Individuals presenting to high volume hospitals were from high incidence areas, more likely to have a positive family history of cancer. But the percentage of positive incisal edge residue was highest in high volume hospitals (6.2%) (**Table 2**).

**Figure 3:**
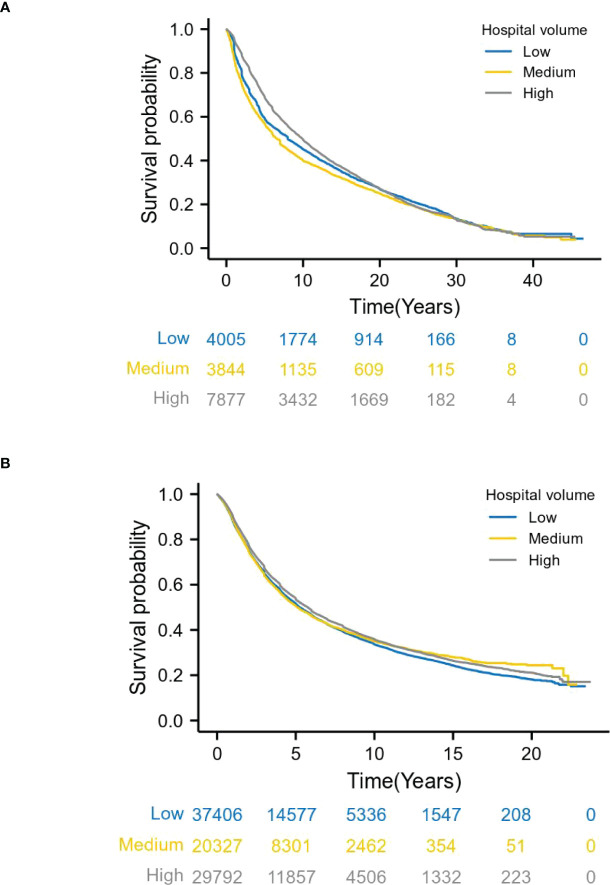
Relationship between hospital volume and overall survival by year. **(A)** shows patients with esophageal cancer from 1973 to 1996, and **(B)** from 1997 to 2020. Long-term survival analysis, Kaplan-Meier curve showed that patients with stage I-III esophageal cancer who underwent surgery in high volume hospitals had better survival than patients in low volume hospitals (log-rank P =0.000).

Long-term survival analysis for 1973-1996 patients and the Kaplan-Meier curve for overall survival demonstrated a survival benefit for treatment at high volume hospitals (log-rank *P* = 0.000). Specifically, patients at medium and high volume hospitals had a reduced risk of death, compared with those at low volume hospitals. The 3-year survival rates in low, medium and large volume hospitals were 66.0%, 61.6% and 75.6%, respectively. The 5-year survival rates were 55.8%, 52.6% and 64.4%, respectively (**Figure 3A**). This trend also existed in patients for diagnosed between 1997 and 2020(log-rank *P* = 0.000)(3-year survival rates: 57.8%, 56.8% and 60.2%; 5-year survival rates:46.8%, 46.4% and 48.8%) (**Figure 3B**).

### Multivariable analysis

**Figure 4:**
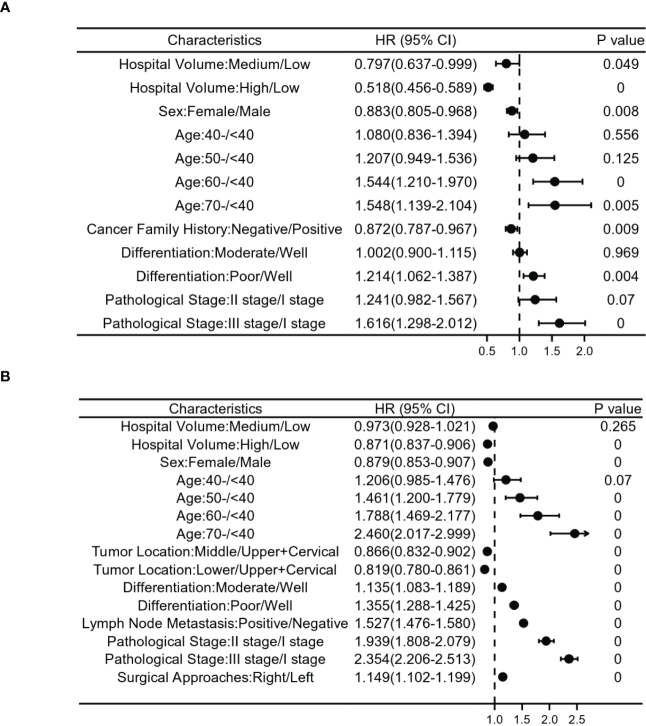
Relationship between clinicopathological features and postoperative survival risk in patients with stages I-III esophageal cancer. **(A)** shows patients with esophageal cancer from 1973 to 1996, and **(B)** ITom 1997 to 2020. Risk ratios based on hospital volume, age, sex, cancer family history, differentiation,tumor location, lymph node metastasis, surgical approaches and pathological stage.

Patients diagnosed between 1973 and 1996 in this study, multivariate analysis demonstrated that after adjusting for patient/tumor-related mixed factors (age, sex, regions, urban/rural residence, cigarette smoking, alcohol consumption, cancer family history, incisal edge residue, tumor location, differentiation and pathological stages), the overall survival rate of medium and high volume hospitals was better than that of low volume hospitals (HR 0.797, 95% Cl 0.637-0.999; HR 0.518, 95% Cl 0.456-0.589). This confirmed the survival benefit of treatment at a high volume hospital. Older age, later pathological stage, poor differentiation, male(HR 0.883, 95%Cl 0.805-0.968), negative family history of cancer (HR 0.872, 95% Cl 0.787-0.967) were associated with a poorer prognosis (**Figure 4A**).

For patients diagnosed between 1997 and 2020, the results of our multivariate Cox proportional hazards model also confirmed the survival benefit of treatment in a high volume hospital. Older age, later pathological stage, poor differentiation, male(HR 0.879, 95%Cl 0.853-0.907) and upper+cervical tumor were independent influencing factors for poor prognosis (**Figure 4B**).

### Hospital volume and all-cause mortality

**Figure 5:**
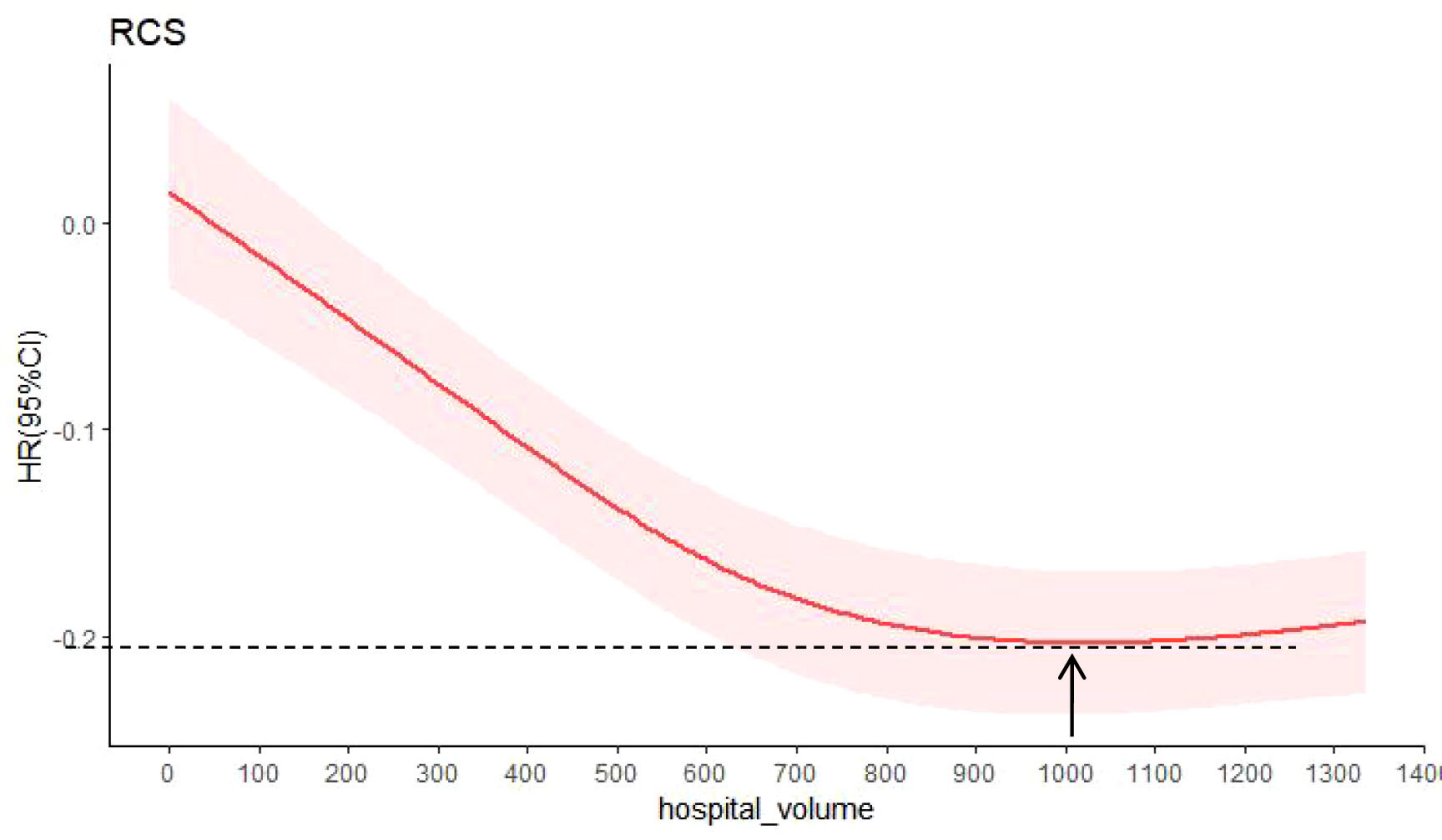
Hazard ratios were multivariable adjusted for death from all causes according to hospital volume. The solid red line is the multivariable adjusted hazard ratio, and the shaded red is the 95% confidence interval obtained from the restricted cubic spline regression. Arrows indicate the hospital volume with the lowest risk of death from all causes. Analyses were adjusted for sex, age, region, urban/rural residence, smoking history, drinking history, cancer family history, incisal edge residue, tumor location, differentiation, pathological stage and contirmed time.

The relationship between hospital volume and the risk of all-cause mortality was half-U-shaped on a continuous scale. However, the overall hospital volume was still a protective factor for postoperative esophageal cancer patients (HR<1). In multivariable adjusted analyses, the hospital volume associated with the lowest risk of all-cause mortality was 1027 cases/year (**Figure 5**).

## Discussion

Based on a large sample size of 158,618 patients spanning 47 years (1973-2020) in China, this paper systematically summarizes the relationship between hospital volume and the treatment effect of ESCC patients with stage I-III in China in two time periods. The hospital volume with the lowest risk of all-cause mortality was found to be 1027 cases/year. These results may provide an important basis for patients to choose hospitals and may have an impact on the centralized management of hospital surgery. As expected, the unlimited increase in hospital volume does not always benefit patients after surgery.

We found that high volume hospitals were both independent predictors of improved survival for stage I-III patients with esophageal cancer in two time periods. Several studies in the United States have shown that treatment in high volume hospitals is better for the long-term survival of patients with esophageal cancer (4, 8, 17–21). Relevant studies in Korea, Switzerland, Australia, Japan and the Netherlands also suggest that centralized surgery for esophageal cancer can improve the clinical prognosis of patients (6, 7, 22–30). However, four other studies in the United States and Sweden found no effect of hospital volume on the postoperative survival of patients with esophageal cancer (9, 10, 31, 32). Our results are consistent with those of most studies. The reason for the inconsistency of our results with those of the Swedish and American studies may be the inclusion of different ethnicities and esophageal cancer subtypes (97% of patients have esophageal squamous cell carcinoma in China, compared with Western countries dominated by esophageal adenocarcinoma). In many tumors and complex procedures, we generally agree that a good survival of patients is strongly associated with hospital volume and the number of thoracic surgeons. Studies have shown that by choosing surgeons who often perform surgery and larger hospitals, patients often can significantly improve their chances of survival (18, 31, 33). Large volume hospitals tend to have better facilities, wider departments and better staffed intensive care units, and other resources, which are not available in small volume hospitals. With these resources, large volume hospitals can better reduce perioperative mortality for cancer patients or high-risk surgical patients (34).

Our results also showed that surgery by the left approach was independent factors of good prognosis in 1997-2020 patients, but not in 1973-1996 patients. One possible explanation for the inconsistent results is that our study included only 7,029 patients with a well-defined surgical approach in the first period (much less than the 41,417 patients in the second period), and the results were statistically biased. It is necessary to enroll more patients with a clear surgical approach for validation. In fact, controversy exists between open esophagectomy by the left approach(Sweet procedure) and surgery by the right approach(Ivor-lewis procedure and Mckeown procedure). In China, left-side approach surgery is the main traditional surgical method, and Sweet procedure is widely used because of its simplicity, speed and relatively small trauma (35, 36). Although it has been criticized for failing to clear or completely clear the upper thoracic lymph nodes (37). In contrast, the right-side approach surgery offers better visualization of the thoracic esophagus, and a skilled surgeon can clean the chest from top to bottom of all lymph nodes. However, the operation time was prolonged and related postoperative complications were increased (38). In this study, the left-side approach was the main operation in both time periods, and in the second time period, the left-side approach was an independent factor influencing the prognosis of patients with ESCC. This suggests that a left-side approach with limited lymphadenectomy remains a priority in China for nearly 20 years. However, as it is popular to perform minimum invasive surgery and postoperative adjuvant therapy in recent years. Minimally invasive surgery is promising with less trauma and fewer complications, but its applicability is limited. In order to better understand the influence of different treatment methods on postoperative prognosis of patients with ESCC, we searched the database for all patients underwent minimally invasive surgery, surgery and surgery + adjuvant therapy in 2014-2015 to analyze their 5-year survival rates, and found that patients underwent minimally invasive surgery had the best survival, followed by surgery and surgery + adjuvant therapy(**Supplementary Figure S1**). This is consistent with the findings of two other studies (39, 40). Therefore, for patients with ESCC, minimally invasive surgery can be preferred if there are indications for minimally invasive surgery. However, no matter it is minimally invasive surgery or open surgery, it is most important to select the treatment approach suitable for the patient based on the patient’s own conditions and ensure the complete resection of the tumor and thorough dissection of the lymph nodes, which will affect the prognosis of the patient.

It is well known that medical equipment of the hospital, the quality of resection and perioperative management of esophageal cancer can also affect patient outcomes. In order to better evaluate the prognosis of patients undergoing esophagectomy in different hospitals, we divided hospitals into tertiary hospitals and secondary hospitals for prognostic analysis according to hospital size, hospital technical level, medical equipment, hospital management level and hospital quality (i.e., hospital grade). The survival of patients undergoing surgical treatment in tertiary hospitals was better than that in secondary hospitals during the period 1973-1996, but the results were reversed in the latter period (**Supplementary Figure S2**). We carefully compared the composition of hospitals with two levels in two periods, and found that although some hospitals were secondary hospitals from 1997 to 2020, their annual operation volume of esophageal cancer had reached the level of high volume hospitals. Because these hospitals are located in the high incidence area of esophageal cancer (Linzhou) and have a large number of patients, the level of thoracic surgery, ICU and anesthesiology departments in the hospitals has been significantly improved. This suggests that it may be necessary to develop specialized cancer hospitals in China.

Hazard ratios were multivariable adjusted for death from all causes according to hospital volume, we found that hospital volume with the lowest risk of death from all causes was 1,027 cases/year. However, the volume threshold of 1,027 cases/year appears to be higher than the high volume definition in previous studies (4, 6–10). It is important to emphasize that half of the annual new esophageal cancer cases are from China (1, 2), and many of the hospitals included in this study were in the high incidence areas of esophageal cancer in China. Our threshold number of cases was objectively determined based on the adjusted correlation between hospital volume and postoperative outcomes. Despite the intuitive appeal of using surgical volume as a predictor and quality measure of surgical outcome, the methodological rigor of many surgical volumetry-outcome studies has been questioned (41). In this study, the number of surgical procedures was not arbitrarily classified, but was based on the Cox hazards model and RCS, and multiple confounding factors were adjusted for data grouping. Therefore, we believe that the average annual hospital operation volume is a reliable predictor of the prognosis of patients with esophageal cancer.

This study is retrospective and has some limitations. First, the AJCC staging system was updated during the large time span of our study data. However, to overcome this limitation, we used the uniform earlier (2002) clinical staging with fewer errors. Second, as with many large data registries, although we checked every medical record, we are not immune to errors in data entry. Finally, the study did not record the average annual ESCC operation volume of each surgeon in the hospital, so it is uncertain whether the difference in hospital volume is caused by the surgeon volume because surgeon experience is also widely believed to be a key factor affecting the prognosis of complex surgery (42–45). However, the medium and high volume thresholds used in this study (>277 cases/year, >689 cases/year and >596 cases/year, >1005 cases/year) are unlikely to be accurate for surgeons with a low annual ESCC volume.

Our findings suggest that high volume hospitals improve long-term survival for patients with stage I–III ESCC and identify hospital volume thresholds with the lowest risk of death from all causes. Therefore, hospital volume can be used as an indicator of the postoperative prognosis of patients with esophageal cancer. It also suggests the importance for health care providers and policy-makers to advocate regionalization or surgical centralization in areas with high mortality.

## Data availability statement

The raw data supporting the conclusions of this article will be made available by the authors, without undue reservation.

## Author contributions

L-DW and L-LL designed and wrote the paper. L-LL, R-HX, M-XW, LS, P-PW, M-MY, J-FH, KZ, W-LH, X-NH, Z-MF, RW, BL, X-ZW, L-GZ, Q-DB, Y-RQ, Z-WC, J-WK, H-JY, LY, J-LR, X-ML performed data collection, interpretation and follow-up. L-LL, X-KZ and XS contributed to data analysis. F-YZ and L-DW revised the manuscript. All authors contributed to the article and approved the submitted version.
