# Supplementary material for: Long-term effect of hospital volume on the postoperative prognosis of 158,618 patients with esophageal squamous cell carcinoma in China
Source: Front Oncol. 2023 Feb 16;12:1056086. doi: 10.3389/fonc.2022.1056086 (PMC9978392; doi:10.3389/fonc.2022.1056086)
Supplement: Supplementary file 1 [file Image_1.pdf]

Supplementary figure 1

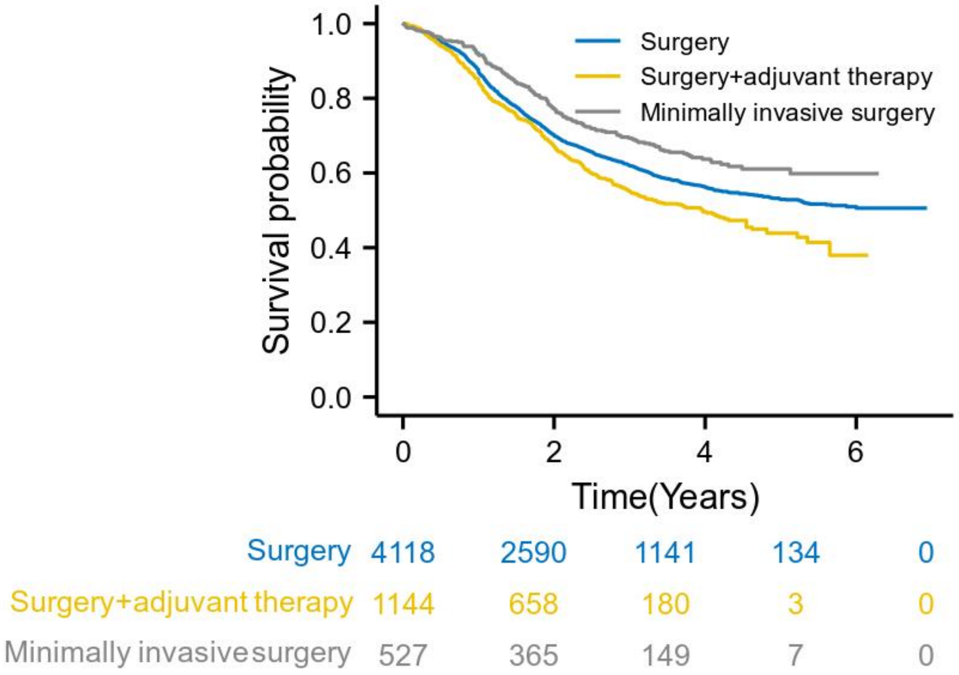

Supplementary figure 1. Relationship between treatment approaches and overall survival by year. The Kaplan-Meier curve showed that patients underwent minimally invasive surgery had the best survival, followed by surgery and surgery + adjuvant therapy (log-rank P =0.000).
