# Supplementary material for: Long-term effect of hospital volume on the postoperative prognosis of 158,618 patients with esophageal squamous cell carcinoma in China
Source: Front Oncol. 2023 Feb 16;12:1056086. doi: 10.3389/fonc.2022.1056086 (PMC9978392; doi:10.3389/fonc.2022.1056086)
Supplement: Supplementary file 2 [file Image_2.pdf]

Supplementary figure 2

A

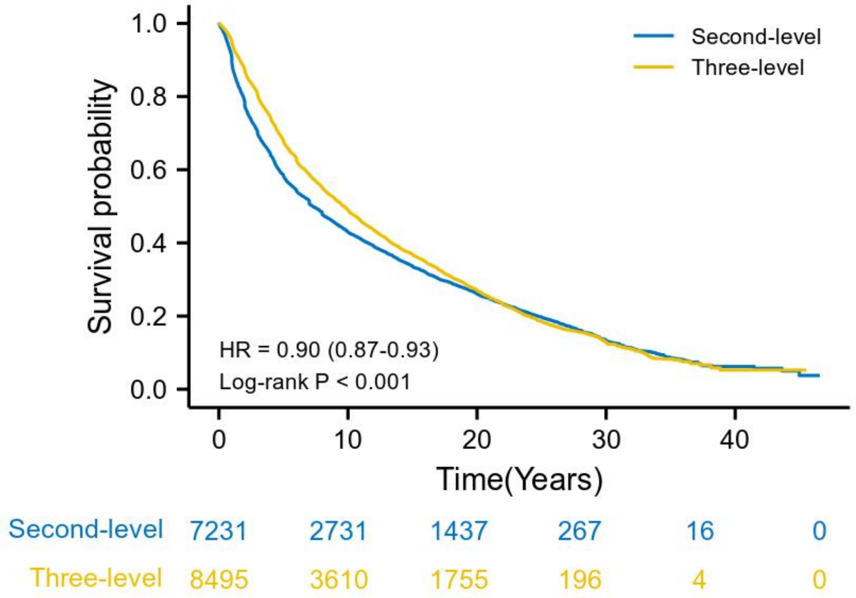

B

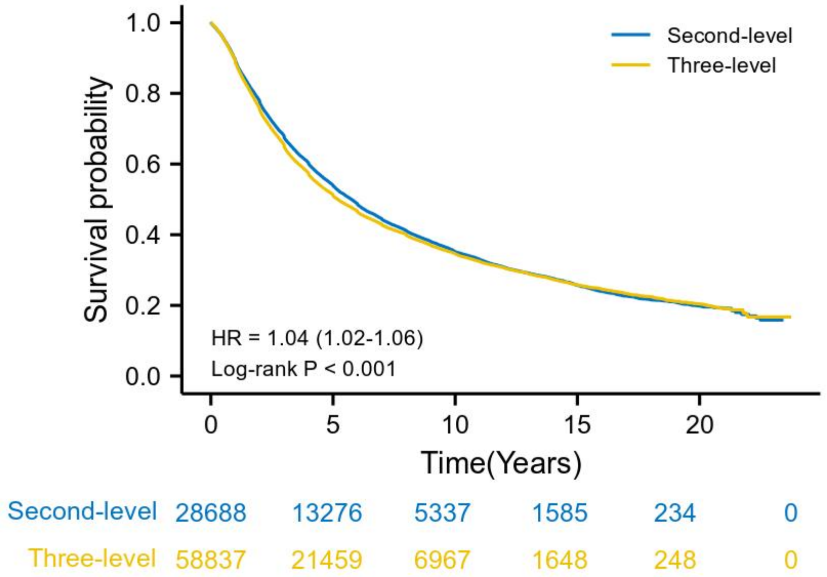

Supplementary figure 2. Relationship between hospital level and overall survival by year. A is the long-term survival analysis of patients with esophageal cancer from 1973 to 1996. The Kaplan-Meier curve shows that patients with stage I-III esophageal cancer who underwent surgery in third-level hospitals had better survival than those in second-level hospitals (log-rank P =0.000). B is the long-term survival analysis of patients with esophageal cancer from 1997 to 2020. The Kaplan-Meier curve shows that patients with stage I-III esophageal cancer who underwent surgery in second-level hospitals had better survival than those in third-level hospitals (log-rank P =0.000).
